# A Unique Case of Cutaneous Schwannoma With Coexistent Masson's Hemangioma

**DOI:** 10.7759/cureus.63600

**Published:** 2024-07-01

**Authors:** Svetlana Bobkova, Eli P Oldham, Igor Shendrik

**Affiliations:** 1 School of Biomedical Sciences, Oklahoma State University Center for Health Sciences, Tulsa, USA; 2 Office of Medical Student Research, Oklahoma State University Center for Health Sciences, Tulsa, USA; 3 Dermatopathology Section, Regional Medical Laboratory, Inc., Tulsa, USA; 4 Dermatopathology Section, Pathology Laboratory Associates, Inc., Tulsa, USA

**Keywords:** immunohistochemistry, vascular proliferative lesion, s100, masson's hemangioma, schwannoma

## Abstract

Schwannomas (SCHs) are benign neural tumors originating from Schwann cells of the peripheral nerve sheaths. These neoplasms typically exhibit hyalinized vessels with impaired vascular permeability; however, angioma-like features are rare. We report an intriguing case of a cutaneous SCH with unusual vascular changes in a 60-year-old female who presented with a tender nodular lesion on her lower back. Histopathological examination of the excised lesion revealed a schwannoma with a central area of thrombosis and a vascular proliferative lesion consistent with Masson's hemangioma (MH). MH, also known as intravascular papillary endothelial hyperplasia (IPEH), is a rare benign vascular lesion characterized by papillary endothelial hyperplasia and obliterative changes within vascular lumens. Immunohistochemical staining confirmed S100 positivity in the SCH component and highlighted the papillary endothelial lining by ERG (erythroblast transformation-specific regulated gene 1). To our knowledge, this is the first report of a schwannoma harboring MH. This unique case underscores the potential for rare vascular proliferation to arise within otherwise typical SCHs.

## Introduction

Schwannomas (SCHs) and Masson's hemangiomas (MHs) are two distinct pathological entities that have not been previously described together. SCHs, also known as neurilemmomas, are benign nerve sheath tumors arising from Schwann cells, which produce the myelin sheath around peripheral nerves [1]. On the other hand, MH, also known as intravascular papillary endothelial hyperplasia (IPEH), is a rare vascular proliferative lesion characterized by the obliteration of vascular lumens due to papillary endothelial hyperplasia and degenerative changes [2]. MH may raise concerns for malignancy due to the presence of interanastomosing vascular channels and endothelial prominence, especially in the small biopsy, limiting architectural evaluation of the lesion [2,3].

## Case presentation

A woman in her mid-60s with an unremarkable medical history presented with a tender nodule on her lower back, measuring 4.3 cm x 3.0 cm. The lesion was longstanding without causing any symptoms, although the exact duration was not documented. However, perceived changes in its size and shape led the patient to seek medical help. She had no prior similar complaints or history of trauma to the generalized area. Upon evaluation by a dermatologist, a preliminary diagnosis of a “right lower back lipoma” was considered, leading to a decision for wide excision to a depth of 1.6 cm.

**Figure 1:**
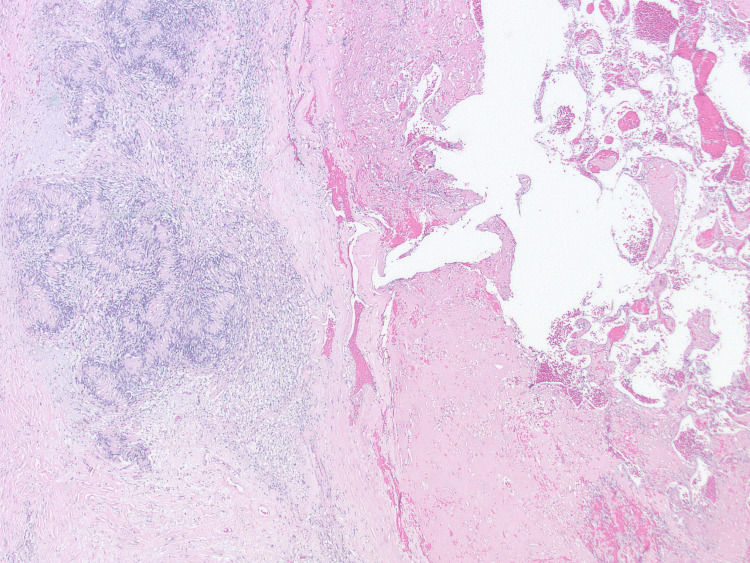
Schwannoma (left part of the image) surrounding the central area of thrombosis and Masson’s hemangioma (right part of the image) (hematoxylin & eosin, original magnification x20).

**Figure 2:**
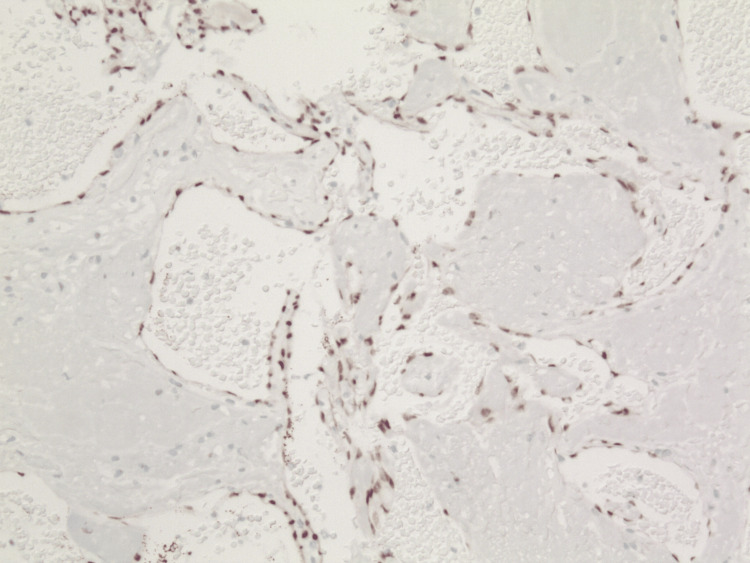
IPEH highlighting a single layer of endothelial cells surrounding papillary structures (ERG, original magnification x100). IPEH: intravascular papillary endothelial hyperplasia; ERG: erythroblast transformation-specific regulated gene 1.

**Figure 3:**
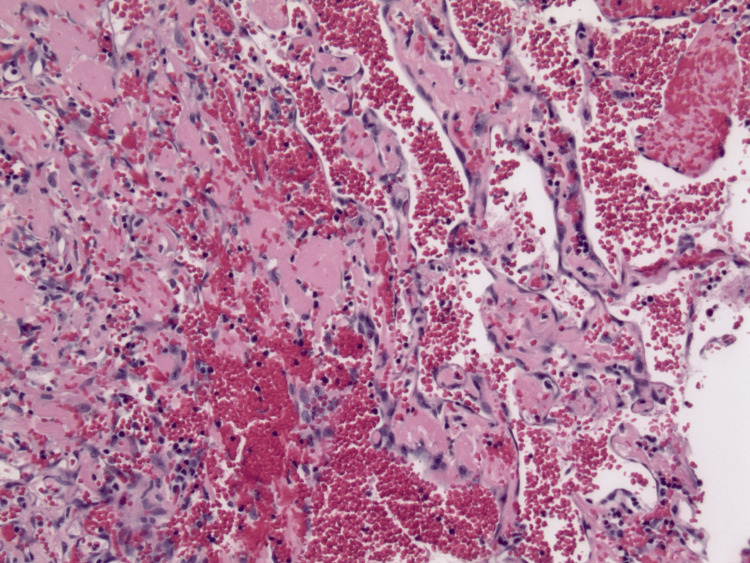
IPEH demonstrating centrally vascularized hyperplasia (hematoxylin & eosin, original magnification x100) IPEH: intravascular papillary endothelial hyperplasia.

Microscopically, the specimen displayed characteristics of both MH and SCH. The biopsy demonstrated an encapsulated nodular lesion with a central area of thrombosis surrounded by cellular spindle cell proliferation (Figure 1). The central thrombotic lesion exhibited areas of papillary fronds lined by a single layer of reactive endothelial cells, highlighted by ERG (erythroblast transformation-specific regulated gene 1) (Figure 2). Interanastomosing vascular channels were noted, but no endothelial multilayering was observed. The areas of vascular hyperplasia were confined to the lesion's center, without an infiltrative growth pattern, which is diagnostic of IPEH (Figure 3).

**Figure 4:**
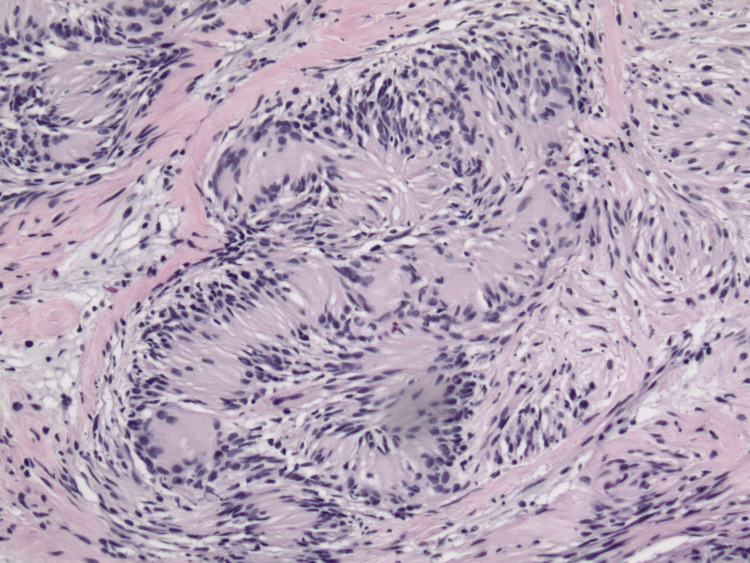
Schwannoma with Verocay body (hematoxylin & eosin, original magnification x100).

**Figure 5:**
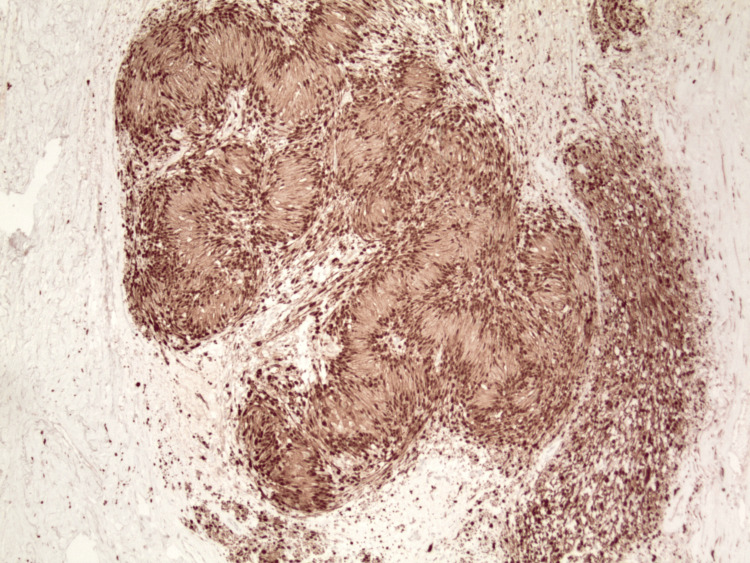
Schwannoma with Verocay body (S100, original magnification x40).

Surrounding the central vascular lesion was a schwannoma, recognized by observable Verocay bodies and a proliferation of elongated spindle cells with wavy nuclei arranged in bundles within a dense fibrillar eosinophilic stroma (Figure 4). The morphologic appearance of schwannoma was confirmed by the lesion's positivity for S100 protein (Figure 5). The IPEH was completely encircled by the SCH. While central areas of the SCH demonstrated myxoid changes, there was no extension of neural proliferation into the areas of IPEH or vascular infiltration into the neural tumor. Melan-A staining was negative in both lesional compartments.

Due to the benign nature of the lesion, excision of the lesion was deemed an adequate treatment for the patient.

## Discussion

SCHs arise from Schwann cells of the peripheral nerve sheaths and are characterized by spindle cell morphology, the presence of Verocay bodies, and a nodular architecture with encapsulation [1]. SCHs also demonstrate prominent vascular spaces, which are frequently hyalinized [1]. These vascular changes are more pronounced in ancient SCHs, which may also exhibit significant anisocytosis and nuclear hyperchromasia [1]. The vascular channels in SCHs exhibit increased permeability, resulting in perivascular hyalinization [1]. Some SCHs show small clusters of vascular spaces, which, although rare, may resemble a vascular tumor [4]. While the presence of intravascular thrombi is seen in SCHs, proliferative vascular changes or papillomatous vascular hyperplasia are not characteristic findings [4].

Masson's hemangiomas, or IPEHs, are a benign intravascular process with features that can mimic other benign and malignant vascular proliferation [2,3]. The characteristic histomorphological feature of IPEH is a vascular lesion consisting of a hyperplastic monolayer of endothelial cells covering a bundle of hyalinized papillary vegetations [2].

The pathophysiology of IPEH is not well understood. The leading hypothesis suggests IPEHs occur from the proliferation of endothelial cells in blood vessels in response to post-traumatic thrombus formation, which is linked to the organization and recanalization of thrombi [5]. Levere et al. proposed an autocrine etiology of post-traumatic IPEH, involving fibroblast growth factor (FGF) secretion [6]. Macrophages at the site of trauma release FGF, which triggers IPEH; the endothelial proliferating cells, in turn, release more FGF, thus activating a positive feedback loop of endothelial proliferation [6].

While IPEH is considered a reactive (non-neoplastic) process, its presentation as a long-standing condition with a relatively high recurrence rate, its appearance in pre-existing vascular tumors, and its GLUT1 negativity and WT1 positivity have led some authors to consider it a true benign neoplasm, possibly beginning as a reactive proliferative process [7].

Current publications classify IPEH into three main subtypes [5]: the primary type (56%), which originates in dilated vascular spaces and presents as a small subcutaneous mass; the secondary type (40%), which develops in an antecedent vascular lesion such as pyogenic granulomas, hemangiomas, or vascular malformations; and the extravascular type (4%), which evolves from a pre-existing hematoma and typically results in larger lesions compared to the other types [8]. The frequent association of IPEH with pre-existing vascular lesions and the absence of a known association of this tumor with neural lesions support the interpretation of the secondary nature of this process within the hemorrhagic and thrombotic area of the SCH.

While the differential diagnosis of IPEH includes benign lesions, such as organizing thrombus, pyogenic granuloma, and arteriovenous malformation, the most clinically important differential diagnosis is with aggressive vascular lesions, particularly angiosarcoma [3]. In this instance, the diagnosis was straightforward due to the complete excision of the lesion, which demonstrated a well-circumscribed vascular lesion surrounded by a neural tumor with classic histologic features of SCH. The centrally located interanastomosing papillary structures lined by ERG-positive cells were limited to the area of the thrombus with no infiltrative vascular proliferation present. The overall lesion architecture, in conjunction with the presence of a thrombus, was diagnostic of IPEH.

While additional immunohistochemical stains are sometimes reported to facilitate the diagnosis of IPEH, markers such as FVIII, type IV collagen, smooth muscle actin (SMA), and muscle-specific actin (MSA) vary widely between individual cases and were deemed noncontributory in this instance [9].

Although it is tempting to suggest a possible pathogenetic link between SCH and IPEH due to the known angiogenic properties of some SCHs, the statistical rarity of such an association suggests it is incidental. Nevertheless, further studies may be necessary to reveal a possible etiopathological link between these tumors.

## Conclusions

This report describes a unique association of SCH surrounding IPEH (also known as Masson's hemangioma). IPEH is a vascular proliferation characterized by interanastomosing vascular channels that must be differentiated from angiosarcoma. Awareness of the possible association between SCH and IPEH may prevent diagnostic confusion and overtreatment, particularly in situations of limited tissue sampling.
